# Recent Insights into the Pathogenesis, Diagnostics, and Treatment of BK Virus Infections in Children After Hematopoietic Stem Cell Transplantation

**DOI:** 10.3390/pathogens14030236

**Published:** 2025-02-28

**Authors:** Mislav Peras, Ernest Bilić, Ivana Mareković

**Affiliations:** 1 Department of Microbiology, Institute of Public Health Zagreb County, 10 000 Zagreb, Croatia; 2 Department of Pediatrics, University Hospital Centre Zagreb, 10 000 Zagreb, Croatia; 3 School of Medicine, University of Zagreb, 10 000 Zagreb, Croatia; 4 Department of Clinical Microbiology, Infection and Prevention Control, 10 000 Zagreb, Croatia

**Keywords:** BK polyomavirus, hematopoietic stem cell transplantation, children, hemorrhagic cystitis, polymerase chain reaction

## Abstract

BK polyomavirus (BKPyV) is a pathogen responsible for infectious complications in hematopoietic stem cell transplant (HSCT) recipients. This review aims to give an insight into recent data about the structure and genomic organization, epidemiology, clinical manifestations, diagnosis, and current treatment options of BKPyV infections in children after HSCT. News regarding viral replication and pathogenesis include the generation of miRNA, new mechanisms of viral shedding by releasing infectious particles via extracellular vesicles, and human bladder microvascular endothelial cells probably acting as viral reservoirs enabling low-level viral replication and persistence. In studies conducted over the past five years, BKPyV hemorrhagic cystitis (BKPyV-HC) has a prevalence rate of 4 to 27% in children undergoing HSCT. Diagnostics still has unsolved dilemmas like whole blood or plasma samples as well as the standardization of molecular methods to allow for reporting in international units. In terms of treatment, new approaches have been used in the past five years, including the use of mesenchymal stem cells (MSCs), virus-specific T cells (VSTs), and recombinant human keratinocyte growth factor (rH-KGF), although the efficacy of some of these treatments has only been documented in isolated studies. This complication continues to pose a substantial clinical challenge, characterized by an absence of effective preventive and therapeutic measures.

## 1. Introduction

Hematopoietic stem cell transplantation (HSCT) is recognized as a therapeutic approach for various malignant and nonmalignant hematologic disorders. While matched-related and matched-unrelated donors are preferred sources of stem cells, many patients, particularly those with aggressive diseases, encounter challenges in finding a suitable donor in a reasonable timeframe [1]. In recent years, there has been a consistent rise in high-risk allogeneic HSCT using human leukocyte antigen (HLA)-mismatched donors, facilitated by improved access to such donors and advancements in graft-versus-host disease (GVHD) prevention [2]. According to the Worldwide Network of Blood and Marrow Transplantation (WBMT), approximately 1.5 million HSCTs were performed globally between 1957 and 2019, with an estimated annual frequency of 84,000 procedures [3].

Recipients of HSCT are highly susceptible to infections due to various factors. The effective prevention and management of infections are critical to ensure the safety and success of HSCT in these vulnerable patients. The administration of cytotoxic chemotherapy leads to conditions like neutropenia, lymphopenia, hypogammaglobulinemia, and damage to mucosal barriers. Additionally, the development of GVHD necessitates the use of immunosuppressive therapies, further compromising the immune response. The presence of invasive catheters also contributes to this heightened risk [4].

Regardless of the recent progress in HSCT, viral infections, in general, continue to be a prevalent cause of morbidity and mortality among HSCT recipients. The significant degree of immune suppression necessary to surpass the HLA barrier is recognized to elevate the risk of potentially severe opportunistic infections caused by double-stranded DNA (dsDNA) viruses, including infection with BK polyomavirus (BKPyV) [5,6]. Post-HSCT patients experience severe lymphopenia, making them highly susceptible to more severe viral infections, including those commonly circulating among healthy children [7]. Therefore, managing viral infections in HSCT recipients is crucial for reducing morbidity and mortality. Viral infections can affect immune reconstitution, disease progression, and GVHD progression, all significantly impacting patient outcomes. Monitoring viral load and virus-specific immune recovery is vital for tailoring antiviral management, while recognizing prognostic factors aids in refining preventive and treatment strategies [8].

BKPyV is a pathogen responsible for infectious complications in transplant recipients. The two major complications associated with BKPyV are BKPyV-associated nephropathy (BKPyVAN) in kidney transplant (KT) recipients and BKPyV-associated hemorrhagic cystitis (BKPyV-HC) in patients undergoing HSCT [9]. In patients who have received other types of solid organ transplants (e.g., heart, lungs, and liver) and in other immunodeficient individuals, these complications occur only sporadically. Also, BKPyV has rarely been reported as the causative agent of infections outside the urinary tract [10]. In recent years, the oncogenic potential of BKPyV and its involvement in cancer development have been the focus of extensive research [11].

BKPyV-HC is a significant complication following HSCT, with its incidence varying based on the type of HSCT, procedural factors, and patient age. While BKPyV-HC significantly impacts post-HSCT morbidity by extending hospital stay and greatly diminishing quality of life, its contribution to increased post-HSCT mortality remains a topic of debate [12].

This review aims to present recent knowledge regarding the role of BKPyV infection in the pediatric population after HSCT. The structure and genomic organization of BKPyV will be explained, followed by a detailed presentation of the epidemiology, clinical manifestations, diagnosis, and current treatment options of BKPyV infections in children after HSCT.

## 2. Taxonomy and Nomenclature of Polyomaviruses

### 2.1. Polyomaviridae

The *Polyomaviridae* is a family of small, non-enveloped viruses that possess closed circular dsDNA genomes of approximately 5 kilobase pairs (kbp). The diverse family of *Polyomaviridae* consists of six genera (*Alphapolyomavirus*, *Betapolyomavirus*, *Gammapolyomavirus*, *Deltapolyomavirus*, *Epsilonpolyomavirus*, and *Zetapolyomavirus*), each exhibiting a limited host range and infecting mammals, birds, and fish [13]. Due to the development of molecular diagnostic methods, new species are continuously being discovered, and today, a total of 117 species have been documented, with 112 species classified across six genera and five additional unclassified species [14]. To date, a total of 14 human polyomaviruses (HPyVs) have been identified [15,16].

The polyomavirus (PyV) names were until recently assigned in a non-systematic manner, usually based on certain characteristics of the newly discovered virus, such as the patient’s initials, the geographical region or institution where the virus was identified, the disease it causes, etc. The International Committee on Taxonomy of Viruses (ICTV) has published the recommendations and standardized the nomenclature of PyVs, specifying that the name of the virus should be based on the host specificity of each polyomavirus species. The name comprises the natural host of the virus, followed by the term “*polyomavirus*” and a number that indicates the order in which the virus was discovered. Accordingly, for PyVs specific to humans, the name comprises the term “*Human polyomavirus*” followed by the respective number (e.g., BK polyomavirus, according to the recommended taxonomy, is named *Human polyomavirus 1*) [17]. This standardization simplifies the identification and classification of PyVs while facilitating the search of relevant literature and communication among researchers about specific PyVs. In addition to the standardization of nomenclature, the ICTV has also established a definition of new polyomavirus species. Species demarcation is based on specific criteria, including sufficient information about the natural host and a genetic distance of more than 15% in the large tumor antigen (LTAg) coding sequence compared to the closest related species. If the genetic distance is less than 15%, additional biological characteristics, such as host specificity, disease association, and tissue tropism, become crucial for distinguishing species [14].

HPyVs are of significant interest to numerous researchers because of their possible oncogenic potential. Merkel cell polyomavirus (MCPyV), JC polyomavirus (JCPyV), BKPyV, and Trichodysplasia spinulosa-associated polyomavirus (TSPyV) have been associated with the potential role in the development of specific malignant tumors [16]. However, current evidence supports only MCPyV as a confirmed human carcinogen since it was detected in the tissues of patients with skin tumors [18,19]. The oncogenic potential of BKPyV and its role in cancer development are still under investigation, although in vitro studies have demonstrated that the LTAg of BKPyV binds to the tumor suppressor proteins p53 and retinoblastoma protein (pRB) [20].

### 2.2. BK Polyomavirus

*Betapolyomavirus hominis*, commonly known as *Human polyomavirus 1* or BK polyomavirus (BK virus, BKPyV, or BKV), belongs to the genus *Betapolyomavirus* [14,21]. The BKPyV was discovered more than 50 years ago when it was isolated in a cell culture from a urine sample of a Sudanese patient who had undergone a KT 3.5 months earlier. The virus was named after the patient’s initials [22]. BKPyV is classified into four genotypes (I, II, III, and IV), along with multiple subtypes identified based on variations in the gene that encodes the major capsid protein VP1 (Ia, Ib1, Ib2, Ic, IVa1, IVa2, IVb1, IVb2, IVc1, and IVc2) [23]. Table 1 highlights the main characteristics of BKPyV and its replication, which are explained in detail further in the text.

## 3. Structure and Replication of BK Polyomavirus

### 3.1. Structure and Genomic Organization of BK Polyomavirus

BKPyV virions measure approximately 45 nm in diameter; they are non-enveloped and possess an icosahedral capsid. The icosahedral capsid comprises 72 capsomers, each consisting of five molecules of the major capsid protein VP1, whereas the minor capsid proteins VP2 and VP3 are situated on the inner surface of the capsid. Each molecule of VP1 interacts with a single molecule of either VP2 or VP3 [25,27]. BKPyV infectious particles contain the viral genome, which is associated with host cell histones (H2A, H2B, H3, and H4), with each genome typically comprising approximately 20 nucleosomes and creating a unique chromatin-like formation known as a “minichromosome” [26,36]. In contrast to cellular chromatin, these “minichromosomes” lack histone H1, a protein crucial for chromatin compaction, implying that they are not highly condensed within the viral particle [26].

The genome of BKPyV is roughly 5 kbp long in the form of circular dsDNA, associated with cellular histones, and organized into three functional domains:

1. The early region, which encodes regulatory proteins;
2. The late region, which encodes capsid proteins;
3. The non-coding control region (NCCR), encompasses the origin of DNA replication (ORI) and the promoter/enhancer regions that regulate transcription of the early and late viral genes [24].

**Figure 1:**
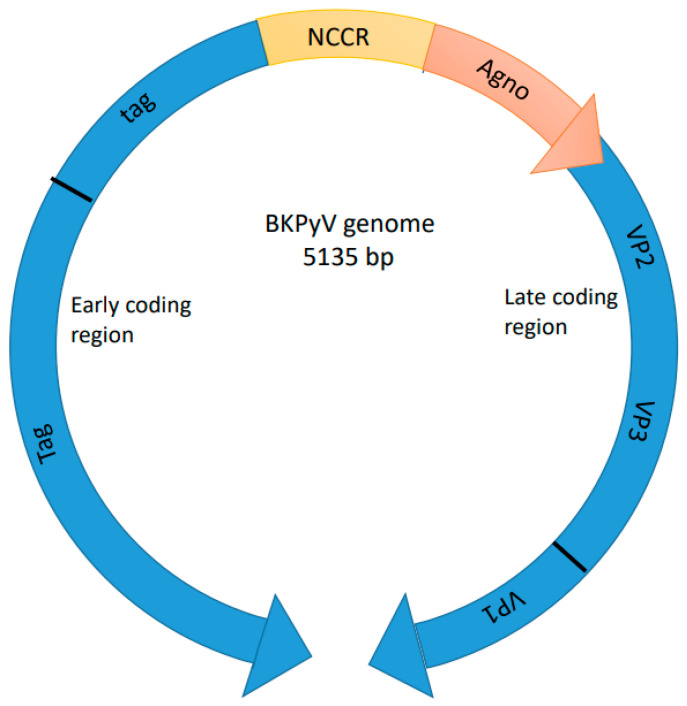
Schematic presentation of BK polyomavirus genome. Abbreviations: BKPyV, BK polyomavirus; NCCR, non-coding control region.

The genes from these different regions have different functions and expression patterns. The early genes encode the LTAg, small TAg (STAg), and truncated large TAg (truncTAg), which are expressed shortly after host cell infection through alternatively spliced messenger RNAs (mRNAs). In contrast, the late genes, expressed after genomic replication begins, encode the structural proteins VP1, VP2, and VP3, along with agnoprotein (Agno). These late proteins are synthesized from two classes of alternatively spliced late RNAs derived from a common pre-mRNA: 19S RNA, which translates into VP2 and VP3, and 16S RNA, which translates into VP1 and Agno [21]. VP1 is the solitary viral protein present on the surface of intact virions; hence, it exclusively mediates the interaction with host cell receptors. Therefore, a monoclonal antibody directed against VP1 offers a promising therapeutic approach to interfere with the virus–host cell contact and avoid the infection of further cells (Figure 1) [37,38].

The functions of BKPyV TAgs have been extensively studied. The LTAg is essential for the initiation of viral replication and interactions with the tumor suppressor proteins p53 and pRB to create a cellular environment conducive for replication. Also, according to a study, the LTAg of BKPyV has an immunoregulatory potential and can interact with different immune cells such as CD4+ and CD8+ T cell subsets [39]. A recent study by Wu et al. found that both interferon alpha (IFNα) and its downstream protein, human myxovirus resistance protein A (MxA), exhibit significant antiviral activity against BKPyV in vitro by suppressing BKPyV LTAg and VP1 synthesis. They also noted that MxA interacted with BKPyV LTAg, inhibiting its nuclear translocation and retaining it in the cytoplasm [40]. On the other hand, a study by Zou et al. investigated the role of STAg and observed that this protein downregulates viral gene expression and DNA replication. Additionally, the study revealed that this process occurs partially through interactions with the subunits of the protein phosphatase 2A (PP2A) enzyme [41].

The function and significance of the truncTAg are less known. Nevertheless, studies reveal that truncTAg is produced from an alternatively spliced mRNA encoding LTAg. TruncTAg is expressed in both BKPyV-transformed and lytically infected cells, with primary localization in the nucleus. The role of truncTAg is probably associated with cell transformation, similar to the supplementary TAgs of other PyVs [42]. Furthermore, Agno interacts with α-soluble N-ethylmaleimide sensitive fusion (NSF) attachment protein (a-SNAP) to regulate nuclear release, and it targets the host’s proliferating cell nuclear antigen protein (PCNA) to downregulate DNA synthesis and cell proliferation. This suggests that Agno may inhibit viral DNA synthesis during the late stages of the viral cycle, facilitating virion assembly. The data indicate that Agno is a key regulator of BKPyV release, contributing to an active egress pathway and supporting a virus-controlled release mechanism [16,43,44].

Numerous studies focus on investigating the function and changes within the NCCR of BKPyV. The NCCR is located between the early and late gene regions, and the transcription of these regions occurs bidirectionally from the ORI situated within the NCCR [45]. Variations in the NCCR distinguish two forms of the BKPyV genome: the archetype and the rearranged form. The archetype, found in the urine of both healthy and diseased individuals, is considered the transmissible form capable of establishing persistent, asymptomatic infection. In contrast, the rearranged form, characterized by deletions or duplications within the NCCR, is associated with disease and is detected in the urine, plasma, and tissues of patients presenting with clinical signs of PyV infection [46,47]. The NCCR has an irreplaceable role in regulating PyV microRNA (miRNA) expression, with research indicating an interesting fact that rearranged BKPyV produces 1000 times more miRNA than the archetype virus. Most PyVs generate viral miRNAs that regulate both viral replication and pathogenesis by targeting viral early genes, as well as host genes [47]. Furthermore, mounting evidence underscores the important role of PyV miRNAs in viral persistence and immune evasion. Since rearranged PyV variants are considered the primary cause of many PyV-related diseases and because these miRNAs behave differently in the replication of the archetype and rearranged variants, further investigation into their biological functions is essential for advancing our understanding of their viral pathogenesis [48].

### 3.2. Replication of BK Polyomavirus in Host Cell

BKPyV interacts with the urothelium and kidney epithelium primarily through the gangliosides GT1b and GD1b, as well as via interactions with alternative b-series gangliosides distinguished by their α-2-8-linked disialyl groups connected to the initial galactose at the reducing terminus (glycosphingolipids containing one or more sialic acid residues) [28]. The attachment of VP1 to host cell receptors is crucial for infection, and several studies describe mutations affecting the major capsid protein VP1 and how they influence attachment to host cells. Variants with specific single-point mutations in the primary receptor-binding loop of VP1 lose the ability to bind host cells and fail to propagate, despite having intact virion structures, DNA packaging, and lytic burst functions [49,50]. Following the attachment to specific viral receptors, the virus enters the host cell through endocytosis. Once inside, it is transported to the endoplasmic reticulum (ER), where the VP1 protein also plays a key role in facilitating entry into the nucleus. BKPyV does not contain a DNA polymerase and requires cellular DNA polymerase to reproduce its genome in the infected cells. Within the nucleus, viral assembly takes place, and the newly formed viral particles are ultimately released through host cell lysis [21,31].

The mechanisms of viral entry into the host cells still remain the focus of extensive research. After binding to receptors, viral particles enter the plasma membrane through invaginations known as caveolae, which are coated with the protein caveolin. Caveolin-dependent endocytosis plays a major role in BKPyV entry into host cells, with gangliosides often being enriched in lipid rafts where this process occurs [30,32]. Caveolin further facilitates endocytosis during BKPyV entry, and the internalized virus is found within vesicles. Additionally, studies have shown that BKPyV enters its natural host cell also via a caveolin-independent and clathrin-independent route. BKPyV then travels along microtubules to the ER lumen, where the capsid partially disassembles. The virus exits the ER via the ER-associated degradation pathway, enters the cytosol, and translocates into the nucleus through the importin α/β pathway [32,51,52,53].

As previously noted, BKPyV shedding typically occurs through cell lysis, where high viral replication causes infected tubular epithelial cells to rupture and exfoliate, appearing as decoy cells in the urine, especially in immunocompromised KT patients with BKPyVAN. However, recent findings suggest an alternative non-lytic shedding mechanism, where infected cells release infectious particles via extracellular vesicles (EVs) [29,54].

### 3.3. Pathogenesis and Role of BK Polyomavirus in Hemorrhagic Cystitis

Initial replication is thought to occur in the respiratory tract, after which the BKPyV spreads via the bloodstream to other organs, particularly targeting renal tubular epithelial and urothelial cells [33]. Within those cells, BKPyV remains in a latent state with the possibility of later reactivation. In KT recipients, the reactivation of latent virus often occurs in response to immunosuppression, resulting in BKPyV infection with three distinct phases: viruria, viremia, and BKPyVAN [55]. Viral proliferation triggers a series of genomic changes that regulate key cellular processes, driving the lytic phase of viral reactivation and allowing the virus to persist in the renal allograft. The primary histopathological alteration noted in BKPyVAN is the infiltration of mononuclear cells within the renal interstitium and tubular epithelial cells. The virus-induced inflammatory response has the potential to cause irreversible kidney damage and allograft injury [56]. Eventually, the viral particles are released through cell lysis, leading to viruria, which subsequently crosses into the interstitium and capillaries, causing viremia. This chain of events culminates in necrosis and the lytic destruction of the renal tubulointerstitium, resulting in inflammation in KT recipients or BKPyV-HC in HSCT recipients [57].

Immunocompromised patients are at risk of uncontrolled BKPyV replication and subsequent graft damage due to several mechanisms. In an immunocompetent individual, intermittent episodes of viral replication activate innate, humoral, and cellular immune responses, which help establish viral control. However, in immunosuppressed transplant recipients, these immune responses are compromised, leading to unregulated viral replication and the development of BKPyVAN, ultimately causing graft damage [58]. To lower the risk of graft rejection, post-transplantation immunosuppressive medications primarily target cellular immunity, which has the most important role in controlling BKPyV infection by preserving viral persistence without replication. This demonstrates that the risk of continuous BKPyV replication is linked primarily to compromised cellular immunity [59].

Several phases have been suggested to explain the pathogenesis of BKPyV-HC among HSCT recipients. Initial bladder mucosa damage is caused by various components of the conditioning regimen. Additionally, the reactivation of latent BKPyV is triggered in urothelial cells, a process further promoted by ongoing immunosuppression, leading to increased BKPyV shedding in urine. Finally, immune reconstitution following engraftment activates immune-mediated urothelial damage (immune reconstitution inflammatory syndrome), which ultimately results in the development of BKPyV-HC [60,61]. BKPyV-HC is categorized into two distinct types according to the timing of its onset: early-onset, which occurs within 72 h, and late-onset, which manifests after 72 h following the conditioning period [62]. Early-onset BKPyV-HC is observed within the first week following the conditioning regimen, while late-onset BKPyV-HC typically occurs during the engraftment phase, generally between the tenth day and six months after HSCT. Early-onset BKPyV-HC is mainly attributed to the toxic effects of conditioning regimens, such as chemotherapy (cyclophosphamide, ifosfamide, etoposide, and busulfan), radiotherapy (pelvic irradiation), or their combination. In contrast, late-onset BKPyV-HC, which develops during engraftment, is thought to result from BKPyV replication in the urinary tract, which has been damaged by previous treatments. This viral replication leads to BKPyV viruria, detectable and quantifiable through molecular diagnostic methods [63,64,65].

Additionally, sustained thrombocytopenia prior to engraftment can contribute to early- and late-onset BKPyV-HC, while coagulopathy or infections with other viruses like JCPyV, cytomegalovirus (CMV), and adenovirus are also often related to late-onset HC [63]. In allogeneic HSCT, conditioning regimens involving bladder-toxic agents such as cyclophosphamide are thought to induce subclinical thinning of the bladder urothelium, which may progress to clinical symptoms due to high-level BKPyV replication and inflammation following engraftment [66]. In certain instances, hemorrhage may become life-threatening, requiring urologic interventions such as cystectomy, and further complications, including renal failure, may lead to a fatal outcome. Therefore, identifying patients at risk is crucial for preventing or intervening the early development of BKPyV-HC [60,67].

### 3.4. BK Polyomavirus Latency, Reactivation, and Oncogenic Potential

Under normal conditions, BKPyV persists in a lifelong latent state within renal tubular epithelial or urothelial cells, typically in an episomal form. However, in immunocompromised individuals, BKPyV can reactivate with high replication levels, leading to complications such as BKPyVAN in KT recipients and BKPyV-HC in HSCT recipients. This latency is thought to be maintained through various regulatory mechanisms involving viral genes, as described in a recent review published by Zhou et al. [35,68].

Firstly, variability in transcription factor binding sites (TFBSs) within the NCCR of the BKPyV archetype can impact viral replication, reactivation, and pathogenesis, enabling the virus to remain latent and potentially reactivate under certain conditions [69]. BKPyV miRNAs contribute to latency by targeting early viral mRNA to suppress replication and by downregulating UL16 binding protein 3 (ULBP3) expression during infection, enabling the virus to evade NKG2D-mediated immune recognition and elimination [70,71]. Moreover, Agno disrupts innate immune signaling by hindering the nuclear translocation of interferon regulatory factor 3 (IRF3), reducing interferon-beta (IFNβ) expression, and altering mitochondrial membrane potential, allowing the virus to evade immune detection and sustain its persistence [72]. Finally, STAg interacts with PP2A to downregulate viral gene expression and DNA replication, a process that likely helps to maintain latency by suppressing active viral replication [41]. Interestingly, studies indicate that renal proximal tubular epithelial cells are immunologically nonresponsive to BKPyV infection. However, human bladder microvascular endothelial cells activate the type I IFN pathway when infected with BKPyV. These immune-responsive cells may act as viral reservoirs, enabling low-level viral replication and persistence [73].

As already mentioned, BKPyV is implicated in tumor development, with several studies and case reports highlighting its oncogenic potential, primarily due to the ability of LTAg to inactivate the pRB and p53 tumor suppressor proteins [68]. A literature review by Iwasaki et al. presented 40 cases of BKPyV-associated urothelial carcinomas that have been reported to date [74]. Also, a recent review by Manole et al. examined the assays used for the identification of oncogenic viruses in renal cell carcinoma (RCC) and urothelial carcinoma and concluded that the most frequently associated virus with these carcinomas was BKPyV [11]. In contrast, a study by Ramqvist et al. investigated the possible association between HPyVs and salivary gland tumors. The analysis of ten different HPyVs, including BKPyV, revealed no significant role for any of them as an etiological factor in these tumors [75]. Although BKPyV-associated carcinomas are rare, they have been observed in KT recipients, with urothelial carcinoma being the most common and RCC reported less frequently. The rising number of documented cases suggests that these tumors may be more prevalent and underdiagnosed among immunosuppressed patients. Among those patients, tumorigenesis is hypothesized to result from the reactivation of latent BKPyV in the urinary epithelium during immunosuppression [76,77].

## 4. Epidemiology

Primary BKPyV infection typically occurs in early childhood, usually before the age of 10, with an average onset between the ages of 4 and 5. It is generally asymptomatic or presents as a mild respiratory illness with minimal clinical significance. Following primary infection, the virus disseminates to the urinary tract, where it remains latent within the urothelium and renal tubular epithelial cells. Viral DNA has also been detected in mononuclear cells, suggesting that BKPyV may infect immune cells and use them to spread from the primary site of infection to latent or persistent sites [34,51]. Maternal antibodies protect newborns from BKPyV infection during the early months of life. However, as these antibodies start to disappear, BKPyV infection begins to emerge, which is reflected in a seropositivity rate of 10% to 30% in infants and increasing from 65% to more than 90% between the ages of 5 and 10 [78]. The seroprevalence of BKPyV in the general population is estimated at approximately 80 to 90% [79,80].

BKPyV infection can be transmitted through several potential routes, including the following:

- Fecal–oral transmission, since the excretion of BKPyV in stool by hospitalized pediatric patients is common [81];
- Airborne respiratory transmission is a possible route, supported by findings of BKPyV DNA in 1% of nasopharyngeal aspirates from newborns with respiratory infections and its detection in tonsillar tissue [81,82];
- Transmission through urine and blood;
- It was noted that BKPyV is likely to be transported with the donor’s kidney to recipients [53,83];
- The vertical transmission of BKPyV has been confirmed through a meta-analysis, showing an estimated 4.9% prevalence and a significantly higher risk of viral reactivation during pregnancy. Although BKPyV has been detected in fetal organs, there is currently no evidence to suggest that it adversely affects pregnant women or their fetuses [84,85].

Variations in the geographical distribution of BKPyV genotypes and subtypes have been observed. Genotype I is the most widespread variant, accounting for 80% of cases globally. Genotype IV is less common (15%) and primarily found in Europe and Asia, while genotypes II and III are considered rare. Subtype Ia is predominantly found in Africa; Ib and Ic are common in Southeast and Northeast Asia, respectively, while Ib2 is widely prevalent in Europe. The subtypes of genotype IV are mainly observed in Asia, except for IVc2, which is more commonly observed in Europe [86,87,88]. A recent study examined the relationship between BKPyV diversity and BKPyV-HC by analyzing 137 urine samples from pediatric HSCT patients. Subtype Ia was the most prevalent, identified in 61.3% of samples (84/137), followed by subtype Ib1 in 31.4% (43/137). While no direct association was found between the BKPyV subtype and BKPyV-HC development, subtype Ia was associated with a higher viral load compared to non-Ia subtypes [89]. However, BKPyV genotyping has been insufficiently explored in many regions, resulting in an unclear geographic distribution of its subtypes. Evidence suggests that certain subtypes may have a greater potential to cause clinical disease and could differ in cellular entry tropism and pathogenicity, but the link between BKPyV diversity and clinical outcomes remains poorly understood [69].

According to the 6th European Conference on Infections in Leukemia (ECIL-6) guidelines, the incidence of BKPyV-HC ranges from 8% to 25% in pediatric patients and 7% to 54% in adult patients, with higher rates observed following allogeneic HSCT compared to autologous HSCT [12]. The lower incidence of BKPyV-HC in autologous HSCT may be due to the absence of immunosuppression and GVHD, factors that play a major role in the pathogenesis of BKPyV-HC in allogeneic HSCT. Unrelated donors are associated with a higher incidence of BKPyV-HC compared to mismatched- or matched-related donors. The incidence is particularly elevated in patients who undergo haploidentical HSCT and receive post-transplant cyclophosphamide (PTCy) as prophylaxis for GVHD [12,90]. In 2024, Zhang et al. conducted a systematic review and meta-analysis to examine the risk factors and incidence of HC, considering both viral and non-viral causes, in pediatric HSCT patients. After analyzing 12 studies involving 2764 participants, they determined that the overall incidence of HC was 9.9%, with 276 cases reported [91]. Based on the studies conducted entirely on the pediatric population over the past five years, BKPyV-HC in children undergoing HSCT has been reported at a rate of 4% to 27%. The estimated rates of BKPyV-HC identified in pediatric HSCT patients, as reported in studies from the past five years, are presented in Table 2.

Studies indicate diversity in the onset of viremia for various viral infections after transplantation, emphasizing the necessity for long-term surveillance on active viral infections in transplant recipients [105,106]. Furthermore, the possibility and occurrence of nosocomial BKPyV infections after HSCT remain uncertain. Some studies evaluated the risk of developing BKPyV-HC in HSCT patients in a hospital setting. The findings suggest that nosocomial transmission of BKPyV may occur post-transplant and should be considered, even though most cases of BKPyV-HC result from the reactivation of a latent virus. These results underscore the importance of implementing strict hygiene measures when managing cases of BKPyV-HC [107,108,109].

### Risk Factors for Development of BK Polyomavirus Hemorrhagic Cystitis

In HSCT, various procedures can affect the incidence of BKPyV-HC, highlighting the importance of understanding and distinguishing between these different procedures. Stem cells for allogeneic HSCT are typically sourced from bone marrow, umbilical cord blood, or peripheral blood. Donors for allogenic HSCT recipients are classified based on HLA matching and may include matched-related donors (MRDs), matched-unrelated donors (MUDs), mismatched-unrelated donors (MMUDs), or haploidentical donors. Moreover, conditioning regimens for allogenic HCT are broadly categorized as myeloablative or reduced-intensity conditioning (RIC), depending on the intensity of the pre-transplant treatment [110].

Over the years, studies have demonstrated that certain risk factors can contribute to an increased incidence of BKPyV-HC in pediatric HSCT recipients. These risk factors may be related to the patient characteristics, the transplant, or the virus itself. Recent studies have identified several of these risk factors in the pediatric population, including male sex [94,96], age > 5 years [93,94,100,101], acute GVHD [92,94,96,97,100,104], the use of a myeloablative conditioning regimen [94,103], the use of total body irradiation (TBI) [94,103], haploidentical donors [96,103], underlying hematologic malignancies (e.g., ALL and AML) [94,100], or urine and blood BKPyV positivity [92,95,97]. A study suggested that the lower incidence of BKPyV-HC in female HSCT recipients compared to males may be attributed to the protective effect of estrogen on the bladder mucosa [111]. Table 2 presents all identified risk factors for developing BKPyV-HC based solely on pediatric population studies published in the last five years. However, a thorough analysis is still required to draw valid conclusions about whether a specific risk factor truly contributes to increased incidence.

Therefore, in 2023, Zhou et al. performed a meta-analysis and systematic review of 11 studies involving 2556 adult and pediatric allogenic HSCT recipients to examine the risk factors for developing only BKPyV-associated HC. This meta-analysis identified that the risk factors significantly associated with BKPyV-HC in the univariable analysis were as follows:

- Patient-related: male sex (odds ratio (OR) = 1.32);
- Transplant-related: haploidentical donor (OR = 1.84), myeloablative conditioning (OR = 1.76), acute GVHD (OR = 2.73), and chronic GVHD (OR = 1.71);
- Virus-related: CMV reactivation (OR = 3.13);
- This meta-analysis identified the use of RIC and MRD as protective factors against BKPyV-HC [112].

Afterwards, in 2024, Zhang et al. performed a meta-analysis and systematic review to examine the risk factors for the development of both non-viral and viral-induced HC in children after HSCT, incorporating 12 studies with a total of 2764 patients evaluated. The identified risk factors for HC in children undergoing HSCT were as follows:

- Patient-related: male sex (OR = 1.52);
- Transplant-related: allogeneic donor (OR = 5.28), HLA-mismatched donor (OR = 1.86), unrelated donor (OR = 1.58), myeloablative conditioning (OR = 3.17), busulfan (OR = 2.18), and anti-thymoglobulin use (OR = 1.65);
- Virus-related: CMV reactivation (OR = 2.64) [91].

Moreover, the same study reported that while RIC was linked to a lower risk of HC, factors such as malignant disease, stem cell source, PTCy or TBI, and the presence of acute or chronic GVHD did not significantly influence the risk for developing HC. Regarding the difference in risk for HC between patients with malignant and nonmalignant diseases, no significant differences were observed; however, in patients with malignant diseases, the intensive chemotherapy required for treatment may damage the bladder mucosa, increasing the occurrence of developing HC after HSCT compared to those with nonmalignant conditions [91].

Regarding the use of PTCy, early-onset HC is commonly related to high-dose Cy, other agents used in conditioning regimens, or GVHD prophylaxis, which cause chemical damage to the bladder mucosa. In the absence of a fully matched donor, allogenic HSCT using haploidentical family donors (haplo-HSCT) has been an alternative for people who do not have human leukocyte antigen (HLA)-matched related or unrelated donors. Some of the strategies in haplo-HSCT, such as the use of PTCy, improved GvHD prophylaxis in patients with malignant and nonmalignant hematologic disorders. The use of cyclophosphamide is associated with many urinary bladder complications like acute hemorrhagic cystitis, chronic cystitis, and other urine abnormalities [113,114].

PTCy use for immunosuppression increases the risk for viral HC. Reports indicate that the incidence of HC in haploidentical HSCT with PTCy ranges from 5.4% to 62%, with an average of approximately 30% [115]. A recent study by Ersoy et al. evaluated the risk factors for BKPyV-HC in pediatric HSCT patients, with a focus on the role of PTCy use. Among 327 patients analyzed, key risk factors identified through multivariate analysis included age (OR: 4.865), the use of TBI, acute GVHD (OR: 2.794), PTCy (OR: 27.353), and CMV co-infection (OR: 2.261). The study revealed a significant association between PTCy and shorter follow-up times to BKPyV-HC onset (*p* < 0.001). Furthermore, the risk for BKPyV-HC increased with the accumulation of multiple risk factors [116]. A study by Apasuthirat et al. compared the immune reconstitution (IR) patterns of 129 children receiving HSCT and analyzed risk factors for viral infection in these patients. It was highlighted that pediatric patients undergoing haploidentical HSCT with PTCy are at a higher risk for BKPyV reactivation and BKPyV-HC than those receiving HLA-matched HSCT. Additionally, patients who received anti-T-lymphocyte globulin (ATG) had significantly lower T-cell counts and a higher rate of blood BKPyV reactivation (40% vs. 15.6%, *p* = 0.019). Specifically, a total of 25 haploidentical HSCT patients developed BKPyV-HC, which was more frequent in the ATG-treated group compared to the non-ATG group (33.3% vs. 15.6%, *p* = 0.087). These findings suggest that haploidentical HSCT with PTCy leads to delayed IR, increasing the risk of BKPyV reactivation, with ATG further aggravating this risk [92].

Finally, a recent study examined the risk factors for severe HC in 77 pediatric HSCT recipients and association with viral co-infections. Key findings indicate that BKPyV infection is a notable independent risk factor for severe HC (HR = 2.782, *p* = 0.035), with its severity exacerbated by the presence of viral co-infections (HR = 2.215, *p* = 0.020). These results highlight the critical role of BKPyV and multi-viral infections in the progression of HC, emphasizing the need for vigilant monitoring and early intervention in high-risk patients [117].

## 5. Clinical Diseases Caused by BK Polyomavirus in Children After Hematopoietic Stem Cell Transplantation

In HSCT patients, BKPyV results in significant morbidity, mainly due to HC. Early HC is mainly related to the conditioning-related toxicity, chemotherapy, radiotherapy, or a combination of these treatments. During the engraftment period, the pathophysiology of HC is primarily influenced by the replication of BKPyV in the urinary tract, which has been previously affected by therapeutic interventions. The involvement of the bladder is characterized by the presence of hematuria, which can manifest with differing degrees of severity (see 6. Diagnostics), and often accompanied by additional symptoms such as dysuria, frequency, urgency, abdominal, and suprapubic pain [94]. In addition to HC, BKPyV viruria may also present as asymptomatic hematuria and interstitial nephritis [100].

In more severe instances, the presence of blood clots may accumulate within the urinary tract, resulting in acute urinary retention, obstructive uropathy, tubulointerstitial nephritis, and ultimately, a decline in renal function. The prevalence of BKPyV-associated nephropathy is still unknown, both in children and adults after HSCT. Renal impairment may arise from a variety of factors, including toxicity associated with conditioning regimens and prophylactic measures for GvHD. Additionally, performing a kidney biopsy in patients who have undergone HSCT carries inherent risks, particularly for those with thrombocytopenia. Namely, kidney biopsies are performed rarely, and therefore, it is difficult to estimate the impact of BKPyV-associated nephropathy on renal impairment after HSCT [65]. In the study by Lee and colleagues, 8 (seven adult and one pediatric case) among 685 HSCT recipients had biopsy-proven BKPyV-associated nephropathy over a 3-year period, with kidney biopsy performed in 14 of the HCT recipients [118].

The role of HC in determining the final outcome is still a matter of debate. Some researchers are reporting a low survival rate, while others have suggested that the clinical course associated with BKPyV-HC is less severe when compared to that caused by ADV. Additionally, researchers indicate that the cause of death should not be directly linked to BKPyV; instead, it is associated with the progression of the underlying condition, bacterial or fungal infections, or GvHD [100].

Less frequently observed clinical manifestations associated with BKPyV infection encompass interstitial pneumonitis and meningoencephalitis [33]. Cases of BKPyV meningoencephalitis have been infrequently documented in HSCT recipients, encompassing cases involving pediatric patients. Clinical manifestations are similar to those found in patients with JCPyV-progressive multifocal leukoencephalopathy (PML), and although they varied including memory loss and a change in mental status, confusion was the most common one [119]. It is assumed that BKPyV can establish latent infection in the central nervous system and be reactivated in the state of immunosuppression in patients after HSCT. The patients have generally poor outcomes [120].

BKPyV-associated pneumonia is infrequently reported in the literature. Most cases develop after or are associated with pulmonary infections caused by other viruses (for example, adenovirus or CMV), bacteria, or fungi [121]. In the assessment of previously described cases of BKPyV-associated pneumonia, the diagnosis relied upon a clinical history that included BKPyV viruria or viremia, HC, or BKPyV-associated nephropathy. Furthermore, the assessment may include the analysis of sputum or bronchoalveolar lavage fluid to detect the presence of BKPyV, in conjunction with imaging techniques such as chest computed tomography (CT) scans or radiographs that exhibit radiological patterns indicative of interstitial pneumonia characterized by ground-glass opacities. With a mortality rate as high as 88.9%, the effectiveness of antiviral drug treatments remains uncertain. A recent report has highlighted a fatal outcome in a pediatric patient after undergoing HSCT [122].

## 6. Diagnostics

### 6.1. Recommendations and Criteria for BKPyV-HC Diagnosis

Viruses are a frequent cause of opportunistic infections in transplant recipients, highlighting the importance of pre-transplant screening, antiviral prevention, and post-transplant monitoring. Real-time polymerase chain reaction (real-time PCR) is a reliable method for detecting viruses, offering high sensitivity, specificity, and the ability to provide qualitative and quantitative data, making it invaluable for disease management and epidemiological studies [105]. Quantifying viral load in plasma/whole blood and urine using quantitative PCR (qPCR) is still the standard diagnostic method for monitoring BKPyV reactivation [33]. For BKPyV, PCR is typically conducted to amplify highly conserved regions within the genes encoding the major capsid protein VP1, LTAg, or genes within NCCR [15].

Based on current data, a review by Saade et al. described the kinetics and phases of BKPyV viruria and viremia in HSCT patients. During the pre-engraftment phase, as BKPyV viruria progressively increases, the virus translocates into the bloodstream through the breakages of peritubular capillaries caused by prior therapies. Once in the blood, BKPyV replicates, and during the engraftment phase, high levels of viral replication can induce urothelial lesions, leading to BKPyV-HC. The virus exploits the damaged urothelium and the immunocompromised state of the host to sustain replication and urinary symptoms. In the post-engraftment phase, as immune reconstitution occurs and mucosal healing progresses, BKPyV viruria gradually declines [65].

In KT recipients, the monitoring of BKPyV replication is already a standard part of clinical practices, with the aim of enabling the early initiation of preemptive therapy and preventing the onset of BKPyVAN [78]. The Second International Consensus Guidelines on the Management of BK Polyomavirus in Kidney Transplantation were published in 2024, proposing recommendations for a routine screening strategy that could enhance clinical outcomes and quality of life in KT recipients. For pediatric KT recipients, monthly screening for plasma BKPyV load is recommended until month 9, followed by screening every 3 months until month 24, with additional screening suggested every 3 months until month 36 post-KT. BKPyV replication is indicated by persistent plasma BKPyV loads of ≥10^3^ copies/mL (assessed on two occasions more than two weeks apart) or a single measurement exceeding ≥10^4^ copies/mL. Urine cytology for decoy cells or urine BKPyV quantification may serve as alternate screening techniques. If urine BKPyV loads are ≥10 million copies/mL or decoy cells are present, plasma BKPyV testing is advised. Upon detection of the aforementioned elevated levels of BKPyV in urine and plasma during routine screening, immunosuppression should be reduced following established protocols. Also, an allograft biopsy may be considered appropriate for confirming BKPyVAN in children, even in the absence of graft dysfunction [123].

The diagnosis of BKPyV-HC is also typically confirmed through PCR testing for BKPyV DNA in urine and plasma/whole blood [4]. In HSCT patients, high-level BKPyV viruria (>7 log_10_ copies/mL) is related to an elevated risk of BKPyV-HC. However, this marker is not exclusive to BKPyV-HC, as approximately 80% of all HSCT patients experience high-level BKPyV viruria, yet only 5–20% develop BKPyV-HC [124]. According to ECIL-6 guidelines, routine screening for BKPyV viruria or viremia in asymptomatic HSCT patients is not recommended outside of clinical studies, as there is currently no effective preemptive treatment for this group of patients available [12]. In contrast to the routine screening of KT patients, it is crucial to highlight that in HCST recipients, PCR testing is conducted based on clinical findings indicative of BKPyV-HC presence. Nonetheless, PCR testing can also detect BKPyV replication even when signs or symptoms of active infection are absent [4,123].

The severity of hematuria, the leading sign of disease, is typically categorized into four grades as follows:

- Grade 1 for microscopic hematuria;
- Grade 2 for macroscopic hematuria;
- Grade 3 for macroscopic hematuria with clots;
- Grade 4 for macroscopic hematuria with clots accompanied by postrenal failure due to urinary tract obstruction [125].

To diagnose BKPyV-HC, according to ECIL-6 guidelines, it is necessary to meet the triad of diagnostic criteria, which includes three essential components as follows:

1. The presence of clinical symptoms or signs of cystitis, such as dysuria and lower abdominal pain;
2. Hematuria, graded as 2 or higher;
3. The detection of BKPyV viruria at levels > 7 log_10_ copies/mL [12].

Recent studies outline several methodologies for detecting patients who may be at risk for developing HC, along with a variety of diagnostic criteria that differ from those provided by ECIL-6. According to Ruderfer et al., urinalysis is conducted for every void during the initial inpatient stay, with microscopic urinalysis performed every 1–2 weeks during the first 100 days after transplantation. Testing for BKPyV is limited to cases with clinical indications such as microscopic or macroscopic hematuria or symptoms suggestive of cystitis [102]. In the study by Yozgat et al., HC was defined as persistent hematuria accompanied by urinary symptoms following the start of conditioning therapy, excluding cases related to gynecological bleeding, systemic bleeding disorders, or urinary tract infections. The onset of HC was marked by the first appearance of symptoms or laboratory evidence after HSCT. For patients presenting with urinary symptoms or hematuria, qPCR testing was conducted on blood and urine samples to detect BKPyV, as well as on blood samples for CMV [99]. A recent review of the literature examining studies published following the establishment of the ECIL-6 criteria has uncovered considerable discrepancies in the definitions and diagnostic criteria employed for the diagnosis of BKPyV-HC in both clinical and research settings. Eleven of the thirty studies analyzed (36.7%) did not clearly specify BKPyV-HC criteria, with eight out of those eleven (72.7%) studies including pediatric patients. Also, only five of the thirty studies (16.7%) analyzed defined BKPyV-HC using the ECIL-6 diagnostic criteria. Notably, only one out of five (20.0%) studies included pediatric patients [126].

### 6.2. Diagnostic Value of PCR Detection in Urine and Blood Samples

According to Cesaro et al., among pediatric patients, a urine BKPyV load of >10^7^ copies/mL showed a sensitivity of 86% and a specificity of 60%, while a plasma BKPyV load of >10^3^ copies/mL demonstrated a sensitivity of 100% and specificity of 86% for detecting HC [127]. Based on the literature, a threshold of 3 log_10_ BKPyV viremia is suggested as an indicator for predicting the development of BKPyV-HC [65]. Plasma viral loads ranging from >3–4 log_10_ copies/mL are observed in over two-thirds of BKPyV-HC cases [12]. In a study by Laskin et al., BKPyV viremia in children monitored during the first 100 days post-HSCT was identified as a significant predictor of BKPyV-HC. Patients without BKPyV viremia were not at increased risk for BKPyV-HC, while those with peak plasma BKPyV loads showed a markedly elevated risk. A peak plasma viral load of 1 to 9999 copies/mL was associated with an adjusted hazard ratio (HR) of 4.2 for developing BKPyV-HC. In contrast, peak viremia exceeding 10^5^ copies/mL resulted in a dramatically higher adjusted HR of 116.8, highlighting the strong correlation between high plasma viral levels and the likelihood of BKPyV-HC [124]. The sensitivity and negative predictive value of BKPyV viremia were revealed to be superior to those of BKPyV viruria in predicting the occurrence of HC [127]. Also, the plasma BKPyV load correlates better with clinical recovery than the urine BKPyV load [128]. In pediatric HSCT recipients, urinary BKPyV DNA loads have been demonstrated to exceed plasma BKPyV DNA loads. Some authors have noted that the sensitivity of detecting BKPyV DNA in blood samples is relatively low, resulting in many patients with HC not showing detectable levels. Although a significant number of HC patients may yield negative results, the presence of BKPyV DNA is often correlated with a negative prognosis. In contrast, urine BKPyV DNA demonstrates a higher sensitivity and is frequently utilized for the early identification of BKPyV-associated HC. It is important to note that while low copy numbers can be found in normal urine, a threshold of more than 10^6^ copies is generally indicative of HC [129]. In a study by Wei et al., 40.9% (61/149) of pediatric HSCT recipients developed BKPyV infection after HSCT. BKPyV DNA was detected in all urine samples and in the plasma of 22 patients. The median BKPyV DNA load was significantly higher in urine (9.50 × 10^7^ copies/mL, range: 5.37 × 10^2^ to 6.84 × 10^9^) compared to plasma (2.97 × 10^3^ copies/mL, range: 9.96 × 10^2^ to 3.58 × 10^8^). BKPyV-HC was diagnosed in 24.2% (36/149) of the BKPyV-infected patients [93]. In a study by Lagala et al., 7% (6/86) of pediatric HSCT recipients developed BKPyV-HC. All six affected patients tested positive for BKPyV in urine, with high-level viruria (>10^7^ copies/mL), whereas BKPyV viremia was detected only in four patients [94]. In a study by Kaya et al., among fifty-one pediatric HSCT patients, three of them who developed BKPyV-HC exhibited urinary BKPyV viral loads of >10^9^ copies/mL within two weeks prior to BKPyV-HC, while a viremia of >10^4^ copies/mL was found in only one patient within two weeks before the onset of BKPyV-HC [104].

It is essential to consider the average onset time of BKPyV-HC, as proactive monitoring of BKPyV viremia during the initial phase may act as a predictive measure for BKPyV-HC. This approach would facilitate the implementation of early interventions, including the reduction in immunosuppression and the provision of supportive care. According to Saade et al., BKPyV viremia in pediatric study populations after HSCT was detected within a median of approximately 10 days after HCT, with BKPyV-HC developing around 15 days later [65]. The onset time of BKPyV-HC among pediatric HSCT patients varied across studies, with Ruderfer et al. reporting a median onset of 37 days (IQR: 26–74) post-HSCT and a median duration of macroscopic hematuria lasting 37.5 days (IQR: 18–71) [102]. Wei et al. noted that BKPyV infection occurred at a median of 23 days (range: 0–273) after the start of conditioning, with urinary BKPyV detected earlier than blood BKPyV [93]. Lagala et al. observed that clinical symptoms, such as abdominal pain, dysuria, macroscopic hematuria, and clots, manifested more than 14 days post-HSCT in all studied cases [94]. Rostami et al. reported a median HC onset of 29 days (IQR: 24–37) post-transplant, with symptoms persisting for a median of 33 days (range: 7–270) [96]. Finally, Salamonowicz-Bodzioch et al. reported a median onset of HC symptoms at 27 days post-HSCT (range: 2–875) [100]. The aforementioned studies, conducted exclusively on the pediatric HSCT population, indicate that the median time for the emergence of BKPyV-HC following HSCT is between 23 and 37 days.

It has been demonstrated that in pediatric HSCT recipients, higher urinary BKPyV DNA levels appear prior to plasma BKPyV DNA levels. Viruria usually precedes BKPyV viremia, which is followed by HC around 11 days later [130]. In a study by Yozgat et al., the median time to the first positive urinary BKPyV DNA was 8 days (range: 1–78), and plasma BKPyV DNA exceeding 10^7^ copies/mL appeared at a median of 13 days (range: 5–92) [99]. In a study by Wei et al., BKPyV in urine was also detected earlier than in plasma samples, with a median time of 13.5 days versus 30.5 days post-transplant (*p* = 0.003), respectively [93].

### 6.3. Plasma or Whole Blood Sample

According to the international guidelines by Kotton et al., plasma is the preferred sample for assessing BKPyV viremia as the standard of care in KT recipients, providing diagnostic value for BKPyVAN [123]. Likewise, the official ECIL-6 guidelines for HSCT recipients include measuring the levels of BKPyV viremia in a plasma sample as a diagnostic criterion for BKPyV-HC [12]. Research on KT recipients indicates that whole blood is less accurate than plasma for PCR detection of BKPyV viremia. In a study by Agrawal et al., 31% (9/29) of KT recipients with biopsy-confirmed BKPyVAN exhibited undetectable BKPyV viremia in whole blood samples. In five patients, BK viremia was identified in retested plasma samples despite being undetected in whole blood [131]. Consequently, whole blood may not be a reliable specimen for detecting BKPyV viremia, whereas the analysis of plasma could demonstrate enhanced sensitivity. Plasma is the only acceptable peripheral specimen for assessing BKPyV viremia in KT recipients. [123,131]. In pediatric HSCT studies, although the ECIL-6 criteria emphasize a plasma sample, the quantification of BKPyV loads is conducted using both whole blood and plasma samples [93,95,101,104]. There are currently no published studies comparing whole blood to plasma samples in HSCT patients.

### 6.4. Current Situation on Standardization of Molecular Methods

The comparability of viral load results between laboratories is challenging due to the absence of a singular reference method for BKPyV DNA quantification. In 2015, the World Health Organization (WHO) constructed the first worldwide standard for BKPyV DNA to serve as the highest-level reference material in clinical diagnostic procedures. These reference standards are quantified in the specific yet arbitrary measure of international units (IU) [132]. Their main goal is to standardize reference materials for routinely used clinical diagnostic assays, providing traceability to a singular common reference preparation irrespective of the methodology employed [133]. The utilization of calibrators based on WHO standards has enhanced interassay variability in quantifying CMV and EBV DNAs. Prior studies also indicate that the use of these standard calibrators has increased concordance among diagnostic assays for identifying BKPyV DNA. However, it has been demonstrated that the WHO standard encompasses subpopulations of BKPyV with diverse deletions in the T region, resulting in variability in PCR results based on the targeted region of the WHO standard [134].The available research on pediatric HSCT patients continues to express the quantification of PCR results for BKPyV DNA in copies/mL. The endorsement of standardized quantitative BKPyV PCR assays, permitting reporting in IU, will enhance the support for transplant patients and facilitate laboratories in making significant comparisons of BKPyV DNA loads. Nonetheless, any calibration technique employed for viral load estimations must include the possible influence of gene rearrangements identified in both calibrants and clinical samples [135]. Therefore, future efforts aim to establish comparable international standards for BKPyV DNA quantification based on defined molecular sequences and the copy numbers of early and late viral gene regions.

### 6.5. Additional Diagnostic Methods

In addition to qPCR, which is the standard method, there are other diagnostic techniques for detecting BKPyV. Digital PCR (dPCR) is a particularly valuable molecular diagnostic method due to its numerous advantages. It is less influenced by PCR inhibitors compared to qPCR methods and delivers highly reproducible results. Additionally, dPCR enables absolute quantification of target sequences without relying on standard curves and offers enhanced analytical sensitivity. This method is particularly effective for monitoring low viral DNA loads, making it a valuable tool for the early detection and management of BKPyV-associated diseases [136,137]. A recent study by Ai et al. analyzed plasma samples from 74 KT patients exhibiting urinary BKPyV-DNA loads surpassing 7 log10 copies/mL utilizing qPCR and droplet dPCR (ddPCR) assays. The limit of detection (LOD) for the ddPCR system was determined to be 100 IU/mL, with a linear range spanning 2.2 to 6.2 log10 IU/mL. Of the 74 clinical samples analyzed, 39 samples tested positive using ddPCR but negative using qPCR. The ddPCR system exhibited high sensitivity (90%) and specificity (67.6%) in detecting low BKPyV-DNA loads in plasma samples, surpassing qPCR, which displayed inferior sensitivity and a higher LOD [136]. However, there are also a few drawbacks of dPCR in comparison to qPCR, including more costly instrumentation and reagents, increased complexity in performance, heightened risk of contamination, reduced throughput, diminished accuracy in quantifying larger amplicons, a narrower dynamic range, a lack of studies in children after HSCT, and restricted multiplexing capabilities [138]. Further investigations will be necessary to ascertain the appropriateness of the WHO IS for ddPCR assays regarding mixed populations of BKPyV being present in the biological standard [132].

Moreover, various tests, including the enzyme-linked immunosorbent assay (ELISA), multiplex immunoassays, and the hemagglutination inhibition test, have been established to identify BKPyV-specific antibodies in patient samples. Most of these assays identify the serological response targeting the immunodominant VP1 capsid protein, while antibodies against the LTAg are rare and present at low titers, rendering them inadequate as infection markers in most instances. However, a standardized and validated test for detecting anti-BKPyV antibodies is not available for commercial use [139].

Research conducted on adult HSCT patients suggests that assessing BKPyV serological status before HSCT may be beneficial in identifying patients at risk for developing BKPyV-HC. Serological testing should be incorporated into the standard pre-HSCT assessment, as antibody levels may correlate with the risk of BKPyV viruria, and measuring subtype-specific antibody titers may suggest a propensity for potential BKPyV reactivation. The information regarding the significance of anti-BKPyV response in donors is also limited. Current findings indicate that in the HSCT setting, the immune response of donors does not have a substantial impact on the reactivation of BKPyV. Furthermore, the serological mismatch between donors and recipients (D−/R+), which has been identified as a significant risk factor for cytomegalovirus reactivation in patients undergoing HSCT, appears to be less influential in the context of BKPyV [140,141]. However, the exact association between BKPyV antibody titer and the risk of developing BKPyV-HC, especially among pediatric HSCT patients, remains unclear. Therefore, it is essential to conduct additional research on this issue. On the other hand, it is important to recognize that post-HSCT antibody levels may not reliably indicate the patient’s serological status due to the significant immunosuppression of both cellular and humoral immunity seen during HSCT. The assessment of BKPyV serological status in post-HSCT patients is of uncertain relevance [141,142]. Thus, qPCR remains the method of choice for assessing and monitoring urine and plasma BKPyV loads in pediatric HSCT patients [4,65].

## 7. Treatment and Prevention

Currently, there is no targeted therapy for symptomatic BKPyV-HC following HSCT. It is essential to implement supportive interventions such as hydration, bladder irrigation, and symptomatic management to reduce pain discomfort. In instances of significant hemorrhaging, substantial transfusion support may be required [130].

From a pharmaceutical standpoint, it is advisable to minimize immunosuppressive therapy whenever feasible; however, this may pose difficulties in patients experiencing active GvHD. The administration of cidofovir could be considered as a potential treatment option; however, there remains ambiguity regarding its effectiveness, optimal dosing regimen, and the necessity to weigh any therapeutic advantages against its renal adverse effects []. Also, antiviral drugs like brincidofovir, as well as immunotherapies like BKPyV-specific T cells are options that could be considered [130]. A small number of uncontrolled studies have yielded successful results regarding non-specific measures promoting the recovery of damaged urothelial epithelium, including hyperbaric oxygen therapy and the application of urologic fibrin glue. Other treatments were also used, but only sporadically, such as intravesical sodium hyaluronate, intravenous FXIII concentrate, leflunomide, oestrogens, and mesenchymal stromal cells [12]. Recombinant human keratinocyte growth factor (rH-KGF) and alprostadil are also options for refractory HC [143,144]. According to some authors, oral leflunomide and oral/intravenous ciprofloxacin are effective in some adult patients but not in children; thus, the treatment options for the affected children are additionally limited [129].

Therapeutic approaches are different across centers worldwide. In order to have an insight into the therapies currently used in the reports published in the last five years, in this review, a search of the literature in PubMed in the period from 2020 to the present using the terms “BK polyomavirus” and “haemorrhagic cystitis”, in combination with the terms “treatment”, “pediatric”, and “children” from 1 January 2020 to 31 December 2024, was made. Reports from 2020 until the present that also included data regarding BKPyV-HC treatment are summarized in Table 3. However, the studies outlined in Table 3 do not primarily focus on evaluating the effectiveness of treatments; rather, they are analyzed to identify the treatment options that have been currently utilized.

In studies performed in the last five years, except supportive treatment and reduced immunosuppression, different types of specific treatment for BKPyV-HC were used. The most common agents used were cidofovir and leflunomide. Their efficacy was evaluated in only one study, with viremia and encephalitis declining and nephropathy remaining resistant to both leflunomide and cidofovir [150].

Current cellular therapies targeting viral infections, known as virus-specific T cells (VSTs), are sourced from either the HSCT donor or a third party. A prevalent method for ex vivo expansion involves stimulating peripheral blood mononuclear cells (PBMCs) with peptide libraries that correspond to the viral antigens of interest. Following this stimulation, T-cell populations that are reactive to the virus are selectively amplified and cultured for 10 to 14 days. The resulting VST product is characterized by a substantial quantity of polyclonal CD4+ and CD8+ T cells, which can target one or multiple viruses concurrently [151]. A recent study by Nelson et al. showed a high rate of response of BKPyV viremia and HC to the VST treatment [147]. CRS has been recorded in ≤2% of patients receiving VSTs [152]. In the research conducted by Holland and colleagues, a fatal consequence of CRS following the administration of VSTs is reported. The authors suggest that the pre-existing inflammatory environment, characterized by elevated levels of BK viremia and complete donor chimerism (100%), along with a heightened reactivity of lymphocytes, likely interacted and collectively led to the severe manifestation of CRS observed in this case [146].

Mesenchymal stromal cells (MSC) possess remarkable self-renewal capabilities and the potential for multidirectional differentiation, which endows them with significant immunomodulatory properties and the ability to facilitate tissue repair. Following intravenous administration, these cells disseminate throughout various tissues and organs. In the presence of an injury or inflammation, they are activated by the cytokines present and migrate back to the affected areas to mediate immune regulation and promote tissue healing. A primary application of MSC therapy in HSCT is the prevention or treatment of GvHD, and many products are used in other diseases besides hematological ones [153]. Thus far, nine of the twelve approved MSC products are from Asia. To date, the European Medicines Agency (EMA) has granted approval to only two MSC therapies. Holoclar is indicated for use in the ocular region, specifically to replace damaged cells in the corneal epithelium of adults suffering from moderate to severe limbal stem cell deficiency resulting from burns. Alofisel has received approval for the management of complex anal fistulas in adult patients diagnosed with Crohn’s disease. In the United States, which has conducted the highest number of MSC clinical trials, the sole approved MSC therapy is Remestemcel-L, an allogeneic cell product utilized for treating steroid-refractory acute GvHD in pediatric patients [154,155]. It is noteworthy that the therapeutic potential of MSCs for acute GvHD appears to be significantly higher in children than in adults. Nevertheless, there is currently an absence of experimental evidence that could shed light on the mechanisms responsible for this disparity. In contrast to numerous other immunosuppressive therapies, there is no indication that MSCs elevate the likelihood of leukemic relapse or the occurrence of opportunistic infections [156]. Over the past five years, there has been only a single study that investigated the use of MSC therapy for severe BKPyV-associated hemorrhagic cystitis (BKPyV-HC). Among thirteen pediatric patients with severe BKPyV-HC, there were eight cured cases and five effective cases, and the total efficacy rate was 100%. Follow-up assessments conducted over a period exceeding three years revealed no significant differences in the infection rates of bacteria, fungi, and viruses between the MSC-treated group and the non-MSC group (Table 3.) [129].

Alprostadil has demonstrated a positive impact in cases that are resistant to other treatments. This compound functions as an agonist for the prostaglandin receptor EP2, which activates adenylate cyclase and leads to an increase in cyclic adenosine monophosphate levels. The resultant effects include the relaxation of smooth muscle, bronchodilation, and a reduction in platelet aggregation. Most patients experience minimal side effects when using intravesical alprostadil; however, earlier research has indicated that bladder spasms occurred in a number of instances [144].

rH-KGF is one of the options for the treatment of refractory BKPyV-HC with only limited data in the literature. In the last five years, only one case report describes its use with a successful outcome. The United States Food and Drug Administration has approved rH-KGF palifermin (Kepivance^®^, Swedish Orphan Biovitrum AB, Stockholm, Sweden) for the treatment of severe oral mucositis in patients undergoing HSCT. Furthermore, it has been employed off-label as a rescue treatment for severe and refractory cases of oral mucositis. rH-KGF exerts its effects by binding to KGF receptors, facilitating the proliferation, differentiation, and migration of epithelial cells. Research has indicated the presence of KGF receptors in several tissues, including the tongue, esophagus, stomach, liver, kidney, and bladder. The effectiveness of rH-KGF in the treatment of BKPyV-HC in children after HSCT has thus far been documented primarily through a limited number of case reports. Additionally, in these reports, the authors have been unable to eliminate the possibility that other concurrently administered therapies may have influenced the positive outcomes observed. Limited case reports by various authors advocate for the use of rH-KGF in instances of severe, refractory hematuria-associated complications (HCs). However, there are theories positing that the administration of rH-KGF may be more advantageous if initiated at an earlier phase, especially at the onset of hematuria, or possibly as a prophylactic intervention in patients at high risk. Additional research beyond these case reports is essential to clarify the therapeutic role of rH-KGF in the management of BKPyV-HC [143].

Information regarding the prevention of BKPyV-HC in pediatric patients following HSCT is limited. The preventive strategy primarily encompasses several key actions: (a) reducing immunosuppression to the extent clinically feasible, given that it is a significant risk factor; (b) initiating early intervention upon the detection of increased viral load or the emergence of symptoms, which may aid in averting the progression to hemorrhagic cystitis; (c) ensuring adequate hydration to facilitate the elimination of the virus from the urinary tract; and (d) providing education to patients and their families, along with regular monitoring, particularly during the initial post-transplant phase, as the prompt identification of symptoms such as hematuria and subsequent medical evaluation are vital for timely intervention.

Currently, there is no licensed vaccine specifically designed for pediatric populations with polyomaviruses. Ongoing research aims to create vaccines that target human polyomaviruses, including BKPyV and JCPyV, which are linked to health issues such as nephropathy in kidney transplant patients and progressive multifocal leukoencephalopathy, respectively. A 2023 study revealed that a multivalent polyomavirus vaccine containing virus-like particles (VLPs) successfully induced long-lasting neutralizing antibody responses in rhesus macaques, indicating promising prospects for future use in humans [157].

## 8. Conclusions

Recent advancements in the understanding of BKPyV have significantly enhanced knowledge regarding its replication, epidemiology, diagnostic methods, and treatment strategies. Research has illuminated the biological mechanisms of BKPyV, particularly highlighting the involvement of miRNA in viral replication and pathogenesis. Additionally, novel pathways of viral shedding have been identified, particularly through extracellular vesicles. The replication of BKPyV appears to be closely associated with compromised cellular immunity, while human bladder microvascular endothelial cells may serve as potential reservoirs for low-level viral replication and persistence. Despite a more comprehensive characterization of BKPyV diversity, the implications of this diversity for clinical outcomes remain inadequately understood. Recent epidemiological studies focusing on BKPyV-HC within pediatric populations over the last five years have reported prevalence rates ranging from 4% to 27%, identifying various risk factors that necessitate careful monitoring and prompt intervention in high-risk individuals. Furthermore, molecular diagnostics continue to face challenges, particularly regarding the use of whole blood or plasma samples, as well as the need for the standardization of molecular techniques through the establishment of international standards for quantification based on specific molecular sequences, facilitating reporting in international units (IU). In terms of treatment, new approaches have emerged in the past five years, including the use of mesenchymal stem cells (MSC), virus-specific T cells (VSTs), and recombinant human keratinocyte growth factor (rH-KGF), although the efficacy of some of these treatments has only been documented in isolated studies.

Significant advancements have been made in understanding the pathogenesis, epidemiology, and risk factors associated with BKPyV-HC; however, this complication continues to pose a substantial clinical challenge, characterized by an absence of effective preventive and therapeutic measures. To address this deficiency, it is essential to develop innovative antiviral treatment strategies, underpinned by thorough clinical trials.

## Figures and Tables

**Table 1:** The main characteristics of BK polyomavirus (BKPyV).

Characteristic	Description Regarding BKPyV
taxonomy	family *Polyomaviridae*; genus *Betapolyomavirus*; species *Betapolyomavirus hominis* or *Human polyomavirus 1*; abbreviations BK virus, BKPyV, BKV [14]
types	genotypes I, II, III, IV; subtypes Ia, Ib1, Ib2, Ic, IVa1, IVa2, IVb1, IVb2, IVc1, and IVc2 [23]
genome organization	approximately 5 kbp; closed circular dsDNA associated with cellular histones (in the form of a “minichromosome”); three domains: early region, late region, and NCCR [24,25,26,27]
capsid composition	non-enveloped, icosahedral structure with 72 capsomers; major capsid protein VP1; minor capsid proteins VP2 and VP3 [25,27]
receptors	gangliosides GT1b and GD1b; glycosphingolipids containing one or more sialic acid residues [28]
viral life cycle	attachment to receptors; entering the cell through caveolin-dependent endocytosis or through caveolin- and clathrin-independent routes; transportation to the ER; entering the nucleus where viral assembly takes place; newly formed viral particles are released through host cell lysis or EVs [29,30,31,32]
tropism	renal tubular epithelial cells and urothelial cells [33]
transmission pathways	fecal–oral; airborne respiratory; through urine and blood; via donor kidney to recipient; vertical transmission [34]
latency mechanisms	STAg downregulates viral gene expression and DNA replication; Agno alters mitochondrial membrane potential and disrupts innate immune signaling; BKPyV miRNAs promote latency by inhibiting viral replication and immune recognition; variability within the NCCR of archetype BKPyV influences replication, reactivation, and latency [35]
associated diseases	BKPyV-associated nephropathy in kidney transplant patients; BKPyV-associated hemorrhagic cystitis in HSCT patients [9]

BKPyV, BK polyomavirus; kbp, kilobase pairs; dsDNA, double-stranded DNA; NCCR, non-coding control region; ER, endoplasmic reticulum; EV, extracellular vesicle; STAg, small tumor antigen; Agno, agnoprotein; miRNA, microRNA; HSCT, hematopoietic stem cell transplantation.

**Table 2:** Rates and risk factors of BKPyV-HC in pediatric HSCT patients in studies published over the past five years.

Study/Year Published/ Reference	Study Years/ Country	Study Design	Number of Pediatric HSCT Patients	Number of BKPyV-HC (%)	Reported BKPyV-HC Risk Factors
Apasuthirat et al. (2024) [92]	2015–2019, Thailand,	Retrospective study	128	22.7% (29/128)	Cytotoxic T cell < 340 cells/cu mm in the first month after HSCT, acute GVHD grades 2–4, urine and blood BKPyV reactivation within 100 days
Wei et al. (2024) [93]	2020–2022, China	Retrospective cohort study	149	24.2% (36/149)	Age > 5 years, use of MMF
Lagala et al. (2024) [94]	2018–2023, Argentina	Descriptive and retrospective study	86	7% (6/86)	Age > 5 years, male sex, acute GVHD, underlying hematologic malignancies (ALL), use of myeloablative conditioning regimen, TBI
Sökmen et al. (2023) [95]	2018–2019, Turkey	Prospective study	51	4% (6/51)	BKPyV positivity before HSCT, acute GVHD
Rostami et al. (2023) [96]	2014–2021, Iran	Retrospective study	200	23% (46/200)	Male sex, haploidentical donors, acute GVHD grade 2–4, chronic GVHD, CMV reactivation
Chen et al. (2023) [97]	2021–2022, China	Retrospective study	247	36.8% (91/247)	Age, incompatible blood types between donors and recipients, acute GVHD, BKPyV urine positivity
Li et al., 2022 [98]	2010–2019, China	Retrospective study	97	26.8% (26/97)	Acute GVHD grade 2–4, mononuclear cells dose
Yozgat et al. (2022) [99]	2010–2017, Turkey	Retrospective study	233	4.2% (10/233)	Non-Determined
Salamonowicz-Bodzioch et al. (2021) [100]	2018–2019, Poland	Prospective multicentric study	133	27% (36/133)	Age > 5 years, peripheral blood transplantation, MUD transplantation, busulfan-cyclophosphamide-melphalan conditioning regimen, AML diagnosis, acute GVHD grade 3–4
Yamada et al. (2021) [101]	2016–2020, Japan	Prospective observational study	74	4% (3/74)	Age > 5 years, female sex
Ruderfer et al. (2021) [102]	2011–2015, United States	Retrospective study	314	21.3% (67/314)	Older age, myeloablative conditioning regimen
Karagun et al. (2021) [103]	2013–2022, Turkey	Retrospective study	334	15% (50/334)	Haploidentical donor, busulfan-cyclophosphamide conditioning regimen, TBI
Kaya et al. (2020) [104]	2015–2017, Turkey	Prospective study	51	5.9% (3/51)	Acute GVHD

BKPyV, BK polyomavirus; HC, hemorrhagic cystitis; HSCT, hematopoietic stem cell transplantation; GVHD, graft-versus-host disease; MMF, mycophenolate mofetil; ALL, acute lymphocytic leukemia; TBI, total body irradiation; CMV, cytomegalovirus; HLA, human leukocyte antigen; MUD, matched-unrelated donor; AML, acute myeloblastic leukemia.

**Table 3:** Treatments used in the reports regarding BK polyomavirus hemorrhagic cystitis in children after hematopoietic stem cell transplantation published from 2020 to the present.

Authors (Year)	Country	Type and Number of Patients Included	Treatment Used	Conclusion Regarding Treatment Efficacy and Recommendation
Sökmen et al. (2023) [95]	Turkey	Fifty-one pediatric patients after allogeneic HSCT. *	For preemptive treatment, the dose of immunosuppressive treatment was reduced; in patients who developed HC, cidofovir, oral levofloxacin, intravenous immunoglobulin, and bladder irrigation were used.	Timely preemptive treatments based on the monitoring of BKPyV viral load is effective in preventing HC.
Salamonowicz-Bodzioch et al. (2021) [100]	Poland	One hundred thirty-three patients who were prospectively tested for BKPyV colonization/infection.	In BKPyV-positive patients aged >4-year without symptoms, oral ciprofloxacin (2 × 10 mg/kg) was administered in the majority of centers; in patients with symptoms, intravenous CDV (5 mg/kg) with probenecid (3 + 1 g or 1.5 + 0.5 g orally depending on body weight and/or age) was used once a week until the absence of clinical symptoms or virus negativity. In very severe HC, intravesicular CDV at the dose of 5 mg/kg/week was used in cases of very severe HC (where intravenous cidofovir was not efficient enough) after 5 doses of CDV or profuse nephrotoxicity; supportive treatments such as analgesia, hyper hydration, forced diuresis, and continuous bladder irrigation were conducted. In cases of significant bleeding and urinary tract obstruction, surgical intervention was performed.	Not evaluated.
Moss et al. (2024) [126]	United States	Case report.	A prolonged course of intravenous CDV followed by leflunomide, keratinocyte growth factor, and hyperbaric oxygen was administered.	No clear response to any of these interventions.
Pfeiffer et al. (2023) [145]	United States	Twenty-seven patients with BKPyV-associated disease.	Posoleucel infusion (a single intravenous infusion of 2 × 10^7^/m^2^ of posoleucel with the option to receive a second infusion after four weeks and additional infusions at biweekly intervals thereafter).	Among the 23 patients who received treatment for refractory BKPyV-associated hemorrhagic cystitis, 74% experienced a resolution of symptoms and macroscopic hematuria within six weeks following the infusion. The administration of posoleucel for the management of refractory viral infections or diseases in recipients of allogeneic HSCT was found to be feasible, safe, and effective.
Karagun et al. (2024) [103]	Turkey	Fifty patients with BKPyV-HC.	Seven pediatric patients were treated only with hydration and supportive treatment. Thirty patients received bladder irrigation with a chondroitin sulfate solution, and forty patients received ciprofloxacin in addition to supportive treatment. Antiviral treatment was administered to 24 patients. Six patients received high-dose cidofovir (5 mg/kg/wk) combined with probenecid, and eighteen patients received low-dose cidofovir (1 mg/kg, 3 times a week). Twelve patients were given hyperbaric oxygen therapy, and six patients were given estrogen therapy combined with hyperbaric oxygen therapy for 30 days as an adjunct to their primary treatment. Coagulation factor Xa was administered to one patient to treat massive bleeding.	Not evaluated.
Tong et al. (2020) [129]	China	Thirteen pediatric patients with severe BKPyV-HC.	In addition to hydration, alkalization, an antiviral treatment, and umbilical cord blood-derived mesenchymal stromal cell (MSC) infusion were provided (1 × 10^6^/kg once a week until the symptoms improved).	Although MSCs do not reduce the number of BKPyV DNA copies in the urine, the cells have a high efficacy rate (100%) and minimal side effects in treating severe BKPyV-HC after unrelated cord blood transplantation among pediatric patients.
Holland et al. (2021) [146]	United States	A 16-year-old male with chemotherapy-associated grade III BKPyV-HC after HSCT for treatment of dedicator of cytokinesis 8 (DOCK8) deficiency.	Cidofovir and two infusions of third-party donor-derived quadrivalent anti-cytomegalovirus (CMV), Epstein–Barr virus (EBV), -adenovirus (ADV), and BKPyV-specific T cells (VSTs) at 5 × 10^7^ cells/m^2^ were administered.	The onset of cytokine release syndrome (CRS), multiorgan failure, and worsening coagulopathy led to hypoxic arrest.
Shamsian et al. (2021) [144]	Iran	A 13-year-old boy diagnosed with acute myeloid leukemia-M4.	The patient was unresponsive to supportive care, leflunomide, intravenous immunoglobulin G, valganciclovir, and alum.	After administrating the first dose of intravesical alprostadil, an acceptable clinical response was observed, and hematuria stopped.
Nelson et al. (2020) [147]	United States	A total of 38 HSCT recipients received BKPyV VSTs. A total of twenty were treated for BKPyV viremia, four for HC, and fourteen for both BKPyV viremia and HC.	Study subjects received a fixed cell dose of up to 5 × 10^7^ VSTs/m^2^ infused intravenously.	Overall response per infusion was 86% for subjects with BKPyV viremia, demonstrating a response to therapy, with 69% achieving a CR (clearing viremia) and 17% achieving PR (a 50% reduction in viremia). All study subjects with HC responded to VST therapy, with 75% achieving CR and 25% PR. In the subgroup of patients who had both viremia and HC, antiviral response was documented in 87% of infusions (58% CR and 29% PR).
Mustafa et al. (2020) [148]	Jordan	A total of 34 patients with B-thalassemia treated with HSCT; HC in one patient.	Platelets transfusion to keep the platelets count more than 100,000/μL, over hydration, bladder irrigation, diuretics	Complete recovery
Akahane et al. (2022) [149]	Japan	Case report: an 18-year-old boy with idiopathic severe aplastic anemia who underwent unrelated HSCT.	Systemic immunosuppressive agents were reduced	Successful treatment
Hughes et al. (2023) [143]	United States	A 16-year-old with grade 3 refractory HC, developed within 24 h after cyclophosphamide completed.	After transfusions, hyperhydration, supportive care with phenazopyridine and oxybutin, intravenous cidofivir, cystoscopy and manual clot evacuation, ciprofloxacin, estrogen, and intravesicular cidofovir-refractory HC, recombinant human keratinocyte growth factor (rH-KGF) was initiated at 60 mcg/kg three times a week based on limited clinical data.	Within 48 h of the first rH-KGF dose, hematuria improved to grade 2, and he became transfusion independent. Over the next 2 weeks, his hematuria improved to grade 1, with full resolution of HC 17 days after his first dose of rH-KGF.
Bush et al. (2020) [150]	United States	A 10-year-old boy with BKPyV viremia, encephalitis, and nephropathy after receiving an allogeneic HSCT for aplastic anemia.	All immunosuppressive therapies were discontinued; oral leflunomide 20 mg daily and IV cidofovir 0.5 mg/kg every 2 weeks (2 doses) with hydration were started; BK viremia persisted and renal function continued to decline; cidofovir was discontinued, but he remained on leflunomide.	While on leflunomide monotherapy with no other immunosuppression, BK viremia persisted but started to gradually decline 28 months post-HSCT and was negative 32 months later; but BKPyV nephropathy remained resistant to multiple anti-BK virus agents, including leflunomide and cidofovir.

* HSCT, hematopoietic stem cell transplantation; HC, hemorrhagic cystitis; CDV, cidofovir; BKPyVAN, BK polyomavirus-associated nephropathy.

## Data Availability

No new data were created or analyzed in this study. Data sharing are not applicable to this article.
